# LISTENING TO THE PATIENTS’ VOICE: A CONCEPTUAL FRAMEWORK OF HIP FRACTURE PATIENTS’ WALKING EXPERIENCE

**DOI:** 10.1093/geroni/igad104.1335

**Published:** 2023-12-21

**Authors:** Laura Delgado-Ortiz, Ashley Polhemus, Alison Keogh, Lynn Rochester, Anja Frei, Milo Puhan, Judith Garcia-Aymerich

**Affiliations:** ISGlobal, Barcelona, Catalonia, Spain; Zürich University, Zürich, Zurich, Switzerland; University College Dublin, Dublin, Dublin, Ireland; Newcastle University, Newcastle upon Tyne, England, United Kingdom; Zürich University, Zürich, Zurich, Switzerland; Zürich University, Zürich, Zurich, Switzerland; IS Global, Barcelona, Catalonia, Spain

## Abstract

Walking is crucial for an active and healthy ageing, but it changes with age and in the presence of diverse health conditions, including hip fracture and frailty. So far, conceptual frameworks of walking have not included the impact of these conditions and individuals’ lived experiences on their walking. Thus, we aimed to identify and synthesize evidence describing the walking experience from the perspective of individuals recovering from a hip fracture, as well as from the perspective of individuals living with highly prevalent walking-impairing conditions of diverse etiology. We conducted a systematic review and meta-ethnography, following appropriate guidance. Out of 2,552 unique records, 117 (8.5% hip fracture) were deemed eligible for the meta-ethnography. We identified seven themes that explain the experience of walking: (1) becoming aware of the walking experience, (2) the walking experience as a link between individuals’ activities and sense of self, (3) the physical walking experience, (4) the mental and emotional walking experience, (5) the social walking experience, (6) the context of the walking experience, and (7) behavioral and attitudinal adaptations resulting from the walking experience. We proposed a framework that describes the interplay between these themes, providing a conceptualization of walking that is grounded in the experiences of individuals recovering from a hip fracture or living with other walking-impairing health conditions, and that may be used to set priorities and improve patient centricity in clinical practice, research and public health initiatives.

